# Retinal displacement using optical coherence tomography angiography and metamorphopsia in eyes undergoing macular hole surgery

**DOI:** 10.1007/s10384-025-01176-5

**Published:** 2025-02-25

**Authors:** Shun Tsukahara, Asuka Takeyama, Masahiro Ishida

**Affiliations:** 1 Department of Ophthalmology, Toho University Graduate School of Medicine, Tokyo, Japan; 2 Department of Ophthalmology, Toho University Ohashi Medical Center, Ohashi, 2-22-36, Meguro-ku, Tokyo, 153-8515 Japan

**Keywords:** Idiopathic macular hole, Optical coherence tomography angiography, Metamorphopsia, Retinal displacement, Macular hole diameter

## Abstract

**Purpose:**

To investigate the relationship between metamorphopsia and retinal structural changes using optical coherence tomography angiography (OCTA) images before and after macular hole (MH) surgery.

**Study design:**

Retrospective, consecutive, case series.

**Methods:**

Twenty-seven eyes of 27 patients undergoing MH surgery with internal limiting membrane peeling were studied. M-CHARTS (MC) was used to evaluate metamorphopsia. Retinal distances were measured between two sets of retinal vessel bifurcations that cross the macula vertically and horizontally near and distal to the macula using OCTA 3x3 mm en face images. The rate of change in retinal distance was defined as retinal displacement%. Basal and minimum MH diameters and retinal thicknesses were measured using Spectralis.

**Results:**

The vertical and horizontal MC scores improved postoperatively (*P*<0.001). The retinal distance decreased in all periods (*P*<0.001), and retinal displacements% was greater near than distal to the center of the macula (*P*<0.001). Retinal displacement% was correlated with basal MH diameter (r_s_=−0.419 to −0.280, *P*<0.001 to 0.045). The horizontal MH diameter was a significant factor related to vertical MC score at baseline and 6 months postoperatively (*P* value range: *P*=0.002–0.004). The rates of change in outer retinal thickness and retinal displacement% near the center of the macula were significant factors related to horizontal MC scores at 6 months (*P*<0.001).

**Conclusion:**

Retinal displacement near the macular region contributes to MH closure after surgery. The larger the MH diameter, the greater the retinal displacement near the macula and the degree of residual postoperative metamorphopsia.

## Introduction

An idiopathic macular hole (MH) is an anatomical crack in the center of the macula that causes visual dysfunctions, including metamorphopsia and aniseikonia, with a focus on micropsia. Advances in surgical techniques have led to vitrectomy with internal limiting membrane (ILM) peeling being the first‒line treatment for MH, which has dramatically improved outcomes, including improved visual acuity and retinal morphology [1–3]. However, even after surgical closure of the MH, residual visual dysfunctions, such as metamorphopsia, bring discomfort to patients in daily life [4].

Recently, changes in retinal structure have been reported to generate metamorphopsia after MH surgery [5–7]. We previously reported that temporal retinal displacement due to ILM peeling in MH surgery contributes to MH closure [8] and that retinal displacement correlates with changes in internal nuclear layer (INL) thickness [9]; these findings led us to form the hypothesis that retinal displacement is caused by optic nerve fiber contraction. We have shown that the displacement of photoreceptor cells is the most important cause of metamorphopsia because the degree of metamorphopsia correlates not only with changes in INL but also with MH diameter [7].

Optical coherence tomography angiography (OCTA) is a noninvasive technique that captures the vascular structure of the macula clearly by obtaining en face images [10, 11]. To better understand the mechanism of metamorphopsia in MH, we investigated the relationship between the degree of metamorphopsia, retinal displacement and retinal structural changes in addition to capturing detailed retinal vessel displacement from OCTA images before and after MH surgery. Specifically, we examined how differences in retinal displacement near and distal to the center of the macula affect the degree of metamorphopsia before and after surgery during the MH closure process.

## Subjects and methods

This was a retrospective case series study that was approved by the ethics committee of Toho University Ohashi Medical Center (H23055_H22046, H23056_22050) and conformed to the tenets of the Declaration of Helsinki. This study was retrospective, and images or patient data were anonymized. Therefore, the ethics committee of Toho University Ohashi Medical Center waived the need for informed consent. Information on this study was presented on our institutional website and all the patients were provided with the opportunity to opt out of this research.

We studied the medical records of 27 eyes of 27 patients who had undergone successful vitrectomy by two vitreoretinal surgeons (M.I. and A.T.) for idiopathic MH at the Department of Ophthalmology, Toho University Ohashi Medical Center, from May 2020 to September 2023 and were followed up for at least 6 months postoperatively. Eyes with ocular complications, such as glaucoma, diabetic retinopathy, macular degeneration, myopic chorioretinal atrophy, rhegmatogenous retinal detachment and corneal diseases, that could affect visual function; eyes with an axial length greater than 27 mm, and eyes that underwent the inverted ILM flap technique were excluded.

Standard 3-port vitrectomy with 25-gauge instruments was performed to repair all MHs. If no posterior vitreous detachment was present after the completion of core vitrectomy, it was created by aggressive aspiration. The ILMs visualized with Brilliant Blue G were grasped with vitreoretinal forceps and peeled to the edge of the vascular arcade in all quadrants in all cases. After air‒fluid exchange, tamponade was performed with 20% sulfur hexafluoride. Patients were instructed to remain in the prone position for one day and then were allowed to assume any body position other than the supine position until the gas disappeared. The phakic eyes underwent cataract surgery simultaneously with vitrectomy.

**Figure 1:**
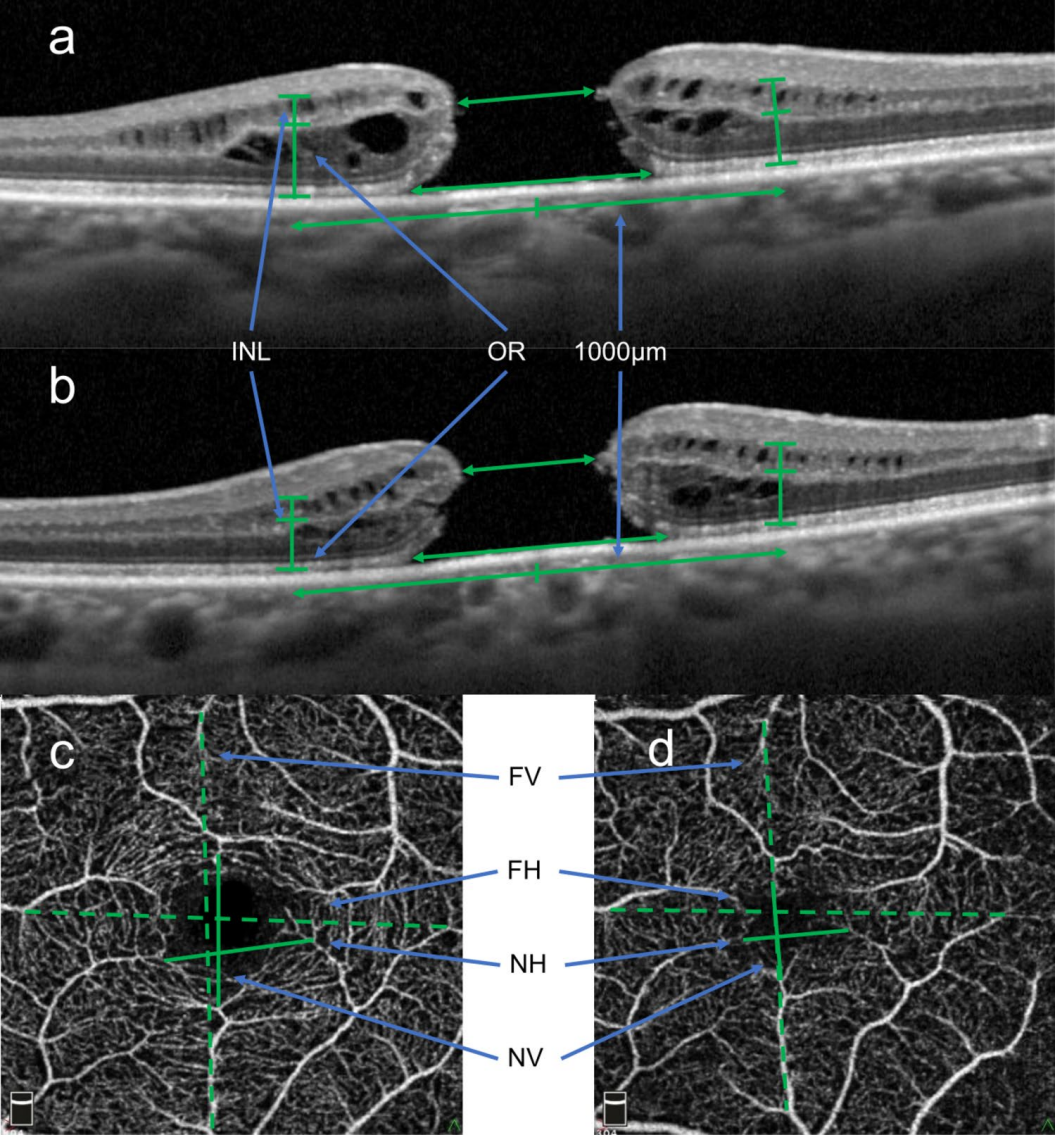
Measurement of optical coherence tomography (OCT) parameters using Spectralis and retinal distances using OCT angiography. **a** In horizontal optical coherence tomography (OCT) scan images, the horizontal minimum and basal diameters of the MH were measured. Inner nuclear layer (INL) and outer retinal layer (OR) thicknesses were measured 1000 μm away from the center of the MH temporally and nasally. **b** In vertical OCT scan images, the vertical minimum and basal diameters of MHs were measured. The INL and OR thicknesses were measured as horizontal images. The right side of the image shows the superior retina, and the left side shows the inferior retina. **c** Preoperative OCT angiography (3x3 mm en face images) was used to measure the distances between the bifurcations of two sets of retinal vessels that cross the macula as vertically or horizontally as possible near and distal to the center of the macula. **d** In en face image taken at 6 months after surgery, the distances between the identical bifurcations of retinal vessels as those measured preoperatively were measured. Solid line; NV: near-vertical retinal distance, NH: near-horizontal retinal distance. Dotted line; FV: far-vertical retinal distance, FH: far-horizontal retinal distance

The basal and minimum MH diameters and retinal thickness were measured using spectral-domain optical coherence tomography (SD-OCT, Spectralis; Heidelberg Engineering). We selected each retinal layer 1000 μm away from the center of the fovea to exclude influence of cystic changes around the fovea [7, 9, 12]. INL thickness and outer retinal layer (OR) thickness in four sectors were measured manually using the caliper function on Spectralis OCT. We calculated the rate of change in thickness for each layer (INL% and OR%). The methods for measuring MH diameter, retinal distance and retinal thickness are shown in Fig. 1-a, b.

OCTA (RTVueXR, AngioVue; Optovue, Inc) 3x3 mm en face images were used to measure pre- and postoperative retinal distances. Retinal distances between the two sets of retinal vessel bifurcations that cross the macula as vertically or horizontally as possible near and distal to the center of the macula were measured (Fig. 1-c, d). In the preoperative images, bifurcations of retinal vessels near the central macula were selected in areas relatively close to the foveal avascular zone (FAZ), whereas bifurcations of retinal vessels distal to the center of the macula were selected at the outside of the ETDRS subfields. The rate of change in retinal distance was defined as retinal displacement%, using the formula: retinal displacement%={(postoperative retinal distance- preoperative retinal distance)/preoperative retinal distance}x100(%). Retinal displacement% [Near-horizontal retinal displacement% (NH%), Near-vertical retinal displacement% (NV%), Far-horizontal retinal displacement% (FH%), Far-vertical displacement% (FV%)] was calculated as the respective rate of change in retinal distance in the near and far regions after surgery.

The best corrected visual acuity (BCVA) was measured using a Landolt C chart at each visit. BCVA was converted to logarithmic (logMAR) units of the angle of minimum resolution for analysis.

M-CHARTS (Inami Co.) was used to evaluate metamorphopsia [13]. Preoperative and postoperative vertical M-CHARTS score (MCV) and horizontal M-CHARTS score (MCH) were measured. Change in metamorphopsia was defined as the difference between pre- and postoperative M-CHARTS scores. All patients underwent examinations at 2 weeks, and 1, 3, and 6 months postoperatively.

Repeated measures ANOVA was used to compare the preoperative and postoperative M-CHARTS scores and retinal distances. The correlations of M-CHARTS score with the MH diameter and retinal displacement% and the correlation of retinal displacement% with the MH diameter were determined by Spearman’s rank correlation test. Paired t-tests were used to compare retinal displacement% at far and near distances. Multiple regression analysis was used to identify the parameters most relevant to metamorphopsia. Statistical analyses were performed using SPSS software version 24.0 (SPSS; IBM Co.,). Differences were considered to be statistically significant at *P* < 0.05.

## Results

The medical records of 27 eyes from 27 patients (male/female: 13 eyes/14 eyes (65.3±6.6 years [mean ± standard deviation]; age range 53–81 years) with idiopathic MH were studied. Eight eyes (29.6%) had stage 2 MH, 11 eyes (40.8%) had stage 3 MH, and 8 eyes (29.6%) had stage 4 MH. Twenty-two phakic eyes underwent phacoemulsification and intraocular lens implantation simultaneously with vitrectomy.

The average minimum MH diameter was 263.6±103.5 μm (range 45–495 μm) in the horizontal direction and 250.5±109.4 μm (range 62–443 μm) in the vertical direction, whereas the average basal MH diameter was 660.0±175.9 μm (range 253–899 μm) in the horizontal direction and 610.5±170.1 μm (range 220–855 μm) in the vertical direction. The average horizontal basal MH diameter was significantly greater than the average vertical MH diameter (*P* < 0.001).

The BCVA was 0.46±0.05 logMAR units preoperatively, unchanged to 0.30±0.05 logMAR units at 2 weeks, and improved to 0.17±0.04 logMAR units at 1 month, 0.10±0.03 logMAR units at 3 months, and 0.04±0.03 logMAR units at 6 months postoperatively (*P* value range: *P* < 0.001–0.060).

### Pre- and postoperative average M -CHARTS scores

**Figure 2:**
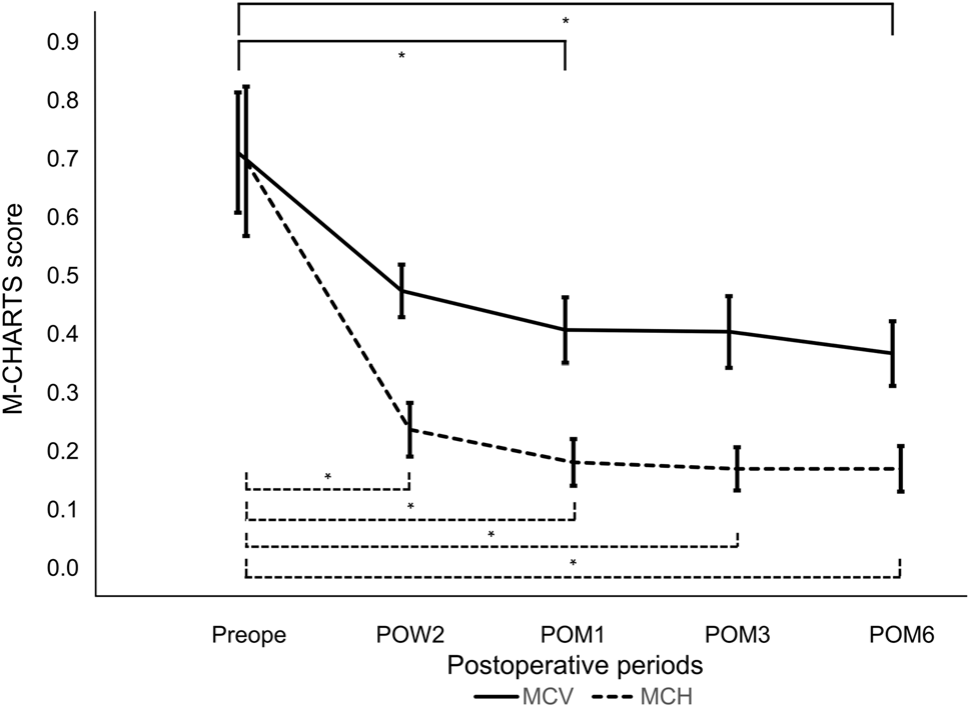
Time course of M-CHARTS scores. The average vertical M-CHARTS score improved significantly at 2 weeks and 6 months postoperatively. The average horizontal M-CHARTS score improved at all postoperative time points (all *P* < 0.001). Solid line; MCV: vertical M-CHARTS score. Dotted line; MCH: horizontal M-CHARTS score. Repeated measures ANOVA was used for comparisons. Significant *P* values are indicated by asterisks (*).

The average preoperative MCV was 0.71±0.10, and 0.47±0.05 at 2 weeks postoperatively (*P* < 0.001), 0.41±0.06 at 1 month, 0.40±0.06 at 3 months, and 0.37±0.06 at 6 months (*P* < 0.001). The average preoperative MCH was 0.70±0.13, improved to 0.24±0.05 at 2 weeks, 0.18±0.04 at 1 month, 0.17±0.04 at 3 months and 0.17±0.04 at 6 months postoperatively (all *P* < 0.001) (Fig. 2).

### Pre- and postoperative average retinal distances

**Figure 3:**
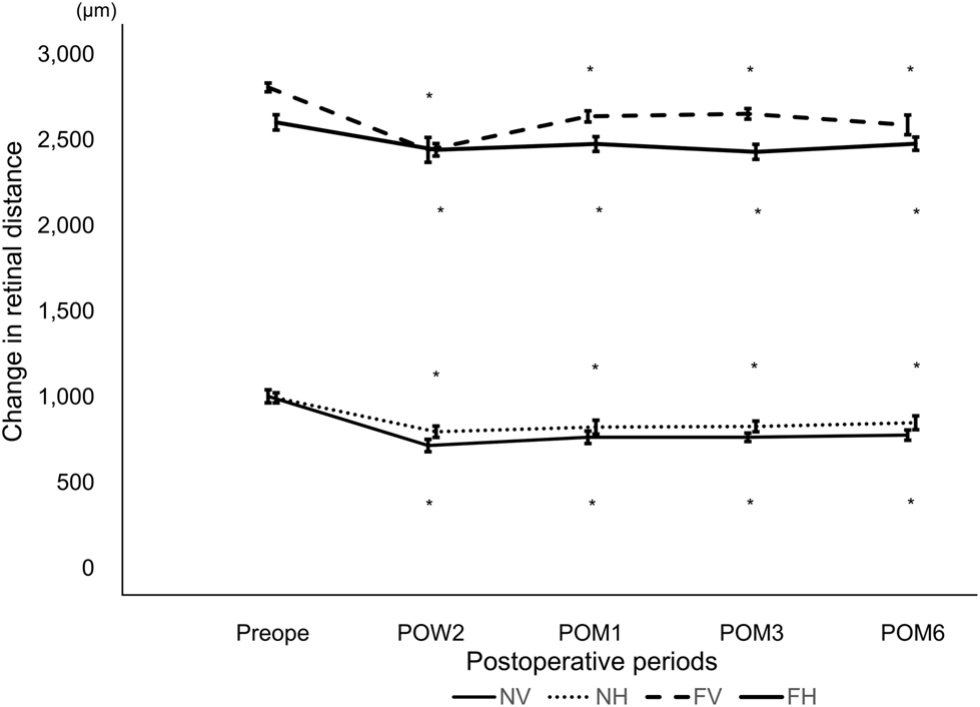
The time course of the retinal distance. Both vertical and horizontal near and far retinal distances were significantly decreased at all visits (all *P* < 0.001). Solid line; NV: near-vertical retinal distance. Dotted line; NH: near-horizontal retinal distance. Broken line; FV: far-vertical retinal distance. Thick line; FH: far-horizontal retinal distance. Repeated measures ANOVA was used for comparisons. Significant *P* values are indicated by asterisks (*).

The average preoperative near-vertical retinal distance was 1003.8±38.2 μm and decreased to 715.5±36.0 μm at 2 weeks, 763.5±36.4 μm at 1 month, 763.5±24.5 μm at 3 months, and 776.46±29.8 μm at 6 months. The average preoperative near-horizontal retinal distance was 994.0±29.5 μm, which decreased to 796.0±33.2 μm at 2 weeks, 823.0±40.5 μm at 1 month, 826.9±31.3 μm at 3 months and 848.5±40.3 μm at 6 months. The average preoperative far-vertical retinal distance was 2806.8±29.5μm and decreased to 2442.0±72.7 μm at 2 weeks, 2637.3±32.6 μm at 1 month, 2652.3±30.9 μm at 3 months, and 2588.0±57.4 μm at 6 months. The average preoperative far-horizontal retinal distance was 2602.4±44.7 μm, which decreased to 2443.0±3.6.5 μm at 2 weeks, 2476.1±43.1 μm at 1 month, 2430.4±43.9 μm at 3 months and 2478.2±38.6.9 μm at 6 months (all *P* < 0.001). The time course of the average retinal distance is shown in Fig. 3.

### Correlations of M-CHARTS scores with the size of macular hole

Preoperative MCH and MCV and those at 6 months did not correlate with minimum and basal MH diameters in any direction, nor did changes in MCH and MCV at 6 months correlate with any MH diameter (r_s_=−0.107 to 0.251, all *P* values > 0.05).

### Correlations of M-CHARTS scores with retinal displacement%

Preoperative MCH and MCV did not correlate with the retinal displacement% at any visit, and MCH and MCV at 6 months and their changes also did not correlate with the retinal displacement% (all *P* values > 0.05).

### Comparison of retinal displacement%

Retinal displacement% was significantly greater in the near region than in the far region at all visits (*P* < 0.001) (Table 1). The vertical retinal displacement% was significantly greater than the horizontal retinal displacement% at 2 weeks, 3 and 6 months in the near region (*P* = 0.008–0.025) and at 2 weeks in the far region (*P* = 0.041).

**Table 1:** Comparison of retinal displacement% in near and far regions

	Retinal displacement%	*P* value		Retinal displacement%	*P* value
2wNV%	−28.80±8.59	*P*<0.001	3MNV%	−23.25±8.29	*P*<0.001
2wFV%	−12.72±13.82		3MFV%	−5.31±3.93	
2wNH%	−19.43±15.48	*P*<0.001	3MNV%	−17.06±11.72	*P*<0.001
2wFH%	−5.97±4.13		3MFV%	−6.32±7.77	
1MNV%	−24.08±7.77	*P*<0.001	3MNV%	−22.45±8.47	*P*<0.001
1MFV%	−5.85±3.47		3MFV%	−7.51±9.20	
1MNH%	−17.59±17.44	*P*<0.001	3MNV%	−15.82±12.54	*P*<0.001
1MFH%	−4.85±4.99		3MFV%	−4.68±4.82	

Paired t-tests were used to determine the significance.

*NV%*: near-vertical retinal displacement%, *NH%*: near-horizontal retinal displacement%

*FV%*: far-vertical retinal displacement%, *FH%*: far-horizontal retinal displacement%

### Correlations of retinal displacement% with basal MH diameter

FV% at 2 weeks correlated with horizontal basal MH diameter. NV% and NH% at 2 weeks and 1 month were correlated with horizontal and vertical basal MH diameters. NH% at 6 months correlated with horizontal and vertical basal MH diameters (Table 2).

**Table 2:** Correlations of retinal displacement% with basal MH diameter.

	Horizontal basal MH diameter		Vertical basal MH diameter	
2wNV%	−**0.342**	*P*=0.014*	−**0.292**	*P*=0.036*
2wFV%	−**0.280**	*P*=0.045*	−0.255	*P*=0.067
2wNH%	−**0.335**	*P*=0.016*	−**0.348**	*P*=0.013*
2wFH%	−0.206	*P*=0.140	−0.194	*P*=0.165
1MNV%	−**0.390**	*P*=0.004*	−**0.316**	*P*=0.021*
1MFV%	−0.151	*P*=0.269	−0.100	*P*=0.466
1MNH%	−**0.419**	*P*=0.002*	−**0.402**	*P*=0.003*
1MFH%	−0.048	*P*=0.723	−0.020	*P*=0.884
3MNV%	−0.128	*P*=0.348	−0.157	*P*=0.252
3MFV%	−0.009	*P*=0.950	0.009	*P*=0.950
3MNH%	−0.208	*P*=0.128	−0.202	*P*=0.139
3MFH%	−0.151	*P*=0.269	−0.145	*P*=0.288
6MNV%	−0.191	*P*=0.162	−0.185	*P*=0.175
6MFV%	0.094	*P*=0.491	0.168	*P*=0.219
6MNH%	−**0.288**	*P*=0.035*	−**0.282**	*P*=0.039*
6MFH%	−0.105	*P*=0.441	−0.054	*P*=0.692

Spearman’s rank correlation coefficient tests were used to determine the significance of the correlations.

*NV%*: near-vertical retinal displacement%, *NH%*: near-horizontal retinal displacement%

*FV%*: far-vertical retinal displacement%, *FH%*: far-horizontal retinal displacement%

Significant *P* values are indicated by asterisk (*). Significant values are in bold (*P*<0.05).

### Multivariate analysis for the M-CHARTS score

Multiple regression analysis was performed to identify a factor that is most relevant to the M-CHARTS scores among the MH diameter, changes in retinal thickness, and retinal displacement%. The objective variables were defined as the MCH and MCV at baseline and 6 months postoperatively, and the explanatory variables were defined as the INL% and OR%, retinal displacement% in near and far regions, and basal MH diameter. For analyses in which the objective variable was the vertical M-CHARTS score, the nasal and temporal rates of change in retinal thickness and horizontal retinal displacement% were selected as the explanatory variables.

As shown in Tables 3 and 4, horizontal MH diameter was a factor most related to the MCV at baseline and at 6 months (*P* value range: *P* = 0.002–0.004). In other words, the larger the horizontal MH diameter, the greater the degree of vertical metamorphopsia before and after surgery.

**Table 3:** Multivariate regression analysis for MCV score at baseline.

Dependent variable	t	β	*P*	95%CI	
				Lower	Upper
Horizontal basal MH diameter	**2.167**	**0.707**	**0.045**	**0.000**	**0.004**
6MIRn%	−0.370	−0.103	0.716	−0.020	0.014
6MINLn%	1.062	0.294	0.303	−0.008	0.024
6MORn%	0.683	0.161	0.504	−0.022	0.042
6MIRt%	−0.690	−0.181	0.500	−0.019	0.010
6MINLt%	−0.867	−0.229	0.398	−0.019	0.008
6MORt%	0.870	0.269	0.397	−0.026	0.062
6MNH%	1.812	0.473	0.088	−0.003	0.044
6MFH%	0.055	0.014	0.957	−0.059	0.062

Independent factors: basal MH diameter, INL%, OR%, RD% in near and far regions.

The nasal and temporal INL% and OR%, and horizontal MH diameter were selected as the explanatory variables.

*RD%*: the rate of retinal displacement at 6 months, *SE*: standard error, *CI*: confidence Interval, *MCV*: vertical M-CHARTS score, *MH*: macular hole, *6MIRn%*: the rate of change in nasal inner retinal thickness at 6 months, *6MINLn%*: the rate of change in nasal inner nuclear layer thickness at 6 months, *6MORn%*: the rate of change in nasal outer retinal layer thickness at 6 months, *6MIRt%*: the rate of change in temporal inner retinal thickness at 6 months, *6MINLt%*: the rate of change in temporal inner nuclear layer thickness at 6 months, *6MORt%*: the rate of change in temporal outer retinal layer thickness at 6 months, *6MNH%*: near-horizontal retinal displacement% at 6 months, *6MFH%*: far-horizontal retinal displacement% at 6 months

Significant values are in bold (*P*<0.05).

**Table 4:** Multivariate regression analysis for MCV at 6 months postoperatively.

Dependent variable	t	β	*P*	95%CI	
				Lower	Upper
Horizontal basal MH diameter	**2.509**	**0.757**	**0.023**	**0.000**	**0.002**
6MIRn%	0.055	0.014	0.957	−0.008	0.009
6MINLn%	0.279	0.072	0.783	−0.007	0.009
6MORn%	−0.283	−0.062	0.780	−0.018	0.014
6MIRt%	−0.721	−0.175	0.481	−0.010	0.005
6MINLt%	0.873	0.214	0.395	−0.004	0.010
6MORt%	1.679	0.481	0.112	−0.004	0.039
6MNH%	1.381	0.334	0.185	−0.004	0.019
6MFH%	−0.239	−0.057	0.814	−0.034	0.027

Independent factors: basal MH diameter, INL%, OR%, RD% in near and far regions.

The nasal and temporal INL% and OR%, and horizontal MH diameter were selected as the explanatory variables.

*RD%*: the rate of retinal displacement at 6 months, *SE*: standard error, *CI*: confidence Interval, *MCV*: vertical M-CHARTS score, *MH*: macular hole, *6MIRn%*: the rate of change in nasal inner retinal thickness at 6 months, *6MINLn%*: the rate of change in nasal inner nuclear layer thickness at 6 months, *6MORn%*: the rate of change in nasal outer retinal layer thickness at 6 months, *6MIRt%*: the rate of change in temporal inner retinal thickness at 6 months, *6MINLt%*: the rate of change in temporal inner nuclear layer thickness at 6 months, *6MORt%*: the rate of change in temporal outer retinal layer thickness at 6 months, *6MNH%*: near-horizontal retinal displacement% at 6 months, *6MFH%*: far-horizontal retinal displacement% at 6 months

Significant values are in bold. *P*<0.05

As shown in Table 5, inferior OR% and NV% at 6 months were factors most related to MCH at 6 months (*P* < 0.001). In other words, the thinner the inferior OR thickness postoperatively and the greater the retinal displacement near the center of the macula, the greater the degree of horizontal metamorphopsia at 6 months postoperatively.

**Table 5:** Multivariate regression analysis for MCH at 6 months postoperatively.

Dependent variable	t	β	*P*	95%CI	
				Lower	Upper
Vertical basal MH diameter	0.400	0.103	0.694	−0.001	0.001
6MIRs%	0.407	0.079	0.689	−0.005	0.008
6MINLs%	−0.062	−0.012	0.951	−0.004	0.004
6MORs%	1.103	0.224	0.286	−0.006	0.020
6MIRi%	0.238	0.050	0.815	−0.005	0.006
6MINLi%	1.746	0.400	0.099	−0.001	0.008
6MORi%	−**2.175**	−**0.454**	**0.044**	−**0.014**	**0.000**
6MNV%	−**2.161**	−**0.395**	**0.045**	−**0.019**	**0.000**
6MFV%	0.619	0.117	0.544	−0.006	0.011

Independent factors: basal MH diameter, INL%, OR%, RD% in near and far regions.

The superior and inferior INL% and OR%, and vertical MH diameter were selected as the explanatory variables.

*RD%: the rate of retinal displacement at 6 months, SE*: standard error, *CI*: confidence Interval, *MCH*: horizontal M-CHARTS score, *MH*: macular hole, *6MIRs%*: the rate of change in superior inner retinal thickness at 6 months, *6MINLs%*: the rate of change in superior inner nuclear layer thickness at 6 months, *6MORs%*: the rate of change in superior outer retinal layer thickness at 6 months, *6MIRi%*: the rate of change in inferior inner retinal thickness at 6 months, *6MINLi%*: the rate of change in inferior inner nuclear layer thickness at 6 months, *6MORi%*: the rate of change in inferior outer retinal layer thickness at 6 months, *6MNV%*: near-vertical retinal displacement% at 6 months, *6MFV%*: far-vertical retinal displacement% at 6 months

Significant values are in bold. *P*<0.05

There were no significant factors for changes in the M-CHARTS score at 6 months (all *P* values > 0.05).

## Discussion

The results of this study show that postoperative retinal displacement near the center of the macula was significantly greater than distal to the macula. Multiple regression analysis revealed that horizontal MH diameter was the most relevant factor to the degree of vertical metamorphopsia both preoperatively and postoperatively. Additionally, the rates of change in OR thickness and vertical retinal displacement near the macula were the most relevant factors to the postoperative horizontal metamorphopsia. In other words, the larger the MH diameter, the greater the retinal displacement near the macula, and the greater the degree of metamorphopsia before and after surgery.

MH formation has been attributed to tangential traction of the prefoveal vitreous cortex or anteroposterior and dynamic macular traction during posterior vitreous detachment [14–16]. Therefore, during MH formation, photoreceptor cells near the center of the macula are efferently displaced along the entire retina, detached from retinal pigment epithelial cells and elevated toward the vitreous cavity. Previously, we demonstrated that during the closure of MHs after vitrectomy with ILM peeling, the retina moves toward the optic disc [8]. As shown in Table 2, the rate of retinal displacement near the center of the macula correlated with the MH diameter. These findings suggest that retinal displacement near the center of the macula may play a significant role in the closure process of the MH. Similar to Akahori et al., the postoperative retinal displacement near the fovea was greater than retinal displacement far from the fovea [17]. However, their study also investigated the effects of postoperative foveal migration by measuring the distance from the fovea. Although the MH diameter was larger horizontally than vertically, the vertical retinal displacement near the macula was significantly greater in the vertical than in the horizontal direction. This occurred because the entire retina around the MH moves toward the optic disc during MH closure, while the superior and inferior retina are displaced afferently; moreover, the temporal retina moves largely towards the center of the macula, whereas the nasal retina moves toward the optic disc, preventing movement toward the center of the macula. These findings suggest that even a relatively small area of ILM peeling around the macula may be sufficient to facilitate retinal mobility and promote MH closure during surgery.

Metamorphopsia resulting from an MH is affected by retinal morphological changes in which photoreceptor cells near the center of the macula are displaced efferently and detach from the retinal pigment epithelium at the edge of the MH [7, 18, 19]. Earlier, we showed that the MH diameter was a predictor of postoperative metamorphopsia [7]. Additionally, retinal structural changes in the photoreceptor layer related to the formation of MHs contributed to the sensation of postoperative metamorphopsia. However, retinal displacement occurring relatively far from the center of the macula did not correlate with the degree of metamorphopsia. Our findings reveal that photoreceptor cells displacement near the center of the macula during MH closure plays an important role in postoperative metamorphopsia. Although the hypothesis behind the mechanism of metamorphopsia has not been histologically proven, it may be supported by clinical evidence. The vascular bifurcation points for the measurement of retinal distance near the central macula were selected relatively close to the boundary with the FAZ. Ando et al. [20] reported that the ratio of the FAZ area of the affected eye to that of the fellow eye did not correlate with metamorphopsia. However, the multiple regression analysis in our study shows that retinal displacement near the macula is the most relevant factor to postoperative metamorphopsia. These findings suggest that greater retinal structural changes near the FAZ may be responsible for residual metamorphopsia postoperatively.

Furthermore, the thinner the OR thickness after surgery, the greater the degree of metamorphopsia at 6 months after surgery. In our previous report [7], MH diameter was an important parameter to determine the degree of metamorphopsia, and the preoperative OR thicknesses were influenced by the size of the MH, of which the baseline OR thickness correlated with the pre- and postoperative metamorphopsia. The thickening of the preoperative OR thickness is considered to reflect the displacement of the photoreceptor layer and the photoreceptor cells near the center of the macula bulging toward the vitreous side during MH formation. The larger MH size indicates greater centrifugal displacement of the photoreceptor cells, which may be slightly displaced from their original position following postoperative MH closure. Although the OR containing photoreceptor cells gradually returns to its original position after surgery and the OR becomes thinner, a slight displacement of photoreceptor cells remains, which may result in residual postoperative metamorphopsia. This indicates that metamorphopsia after MH surgery was caused by irregularities and eccentric displacement of the photoreceptor layer [7, 18].

There are several limitations to this study. This was a retrospective investigation, in which the retinal distance and thickness were measured manually, the ILM peeling area was not quantified, and the retinal displacement following MH surgery resulted in different measurement points for each retinal thickness throughout the preoperative and postoperative periods. Although some statistically significant results were shown in this study, some differences might be due to chance by small sample size. While the analysis was conducted using similar parameters as in our previous report [7], it has been enhanced by the introduction of OCTA, providing a more detailed evaluation of retinal displacement and providing a fresh perspective on metamorphopsia.

In conclusion, retinal displacement near the macular region appears to contribute the most to the MH closure after surgery, as indicated by the measurement of retinal displacement with retinal microvascular structures using OCTA. The results of this study could provide evidence that even a small area of ILM peeling around the macula may be sufficient to enhance retinal mobility and promote MH closure during surgery. The larger the MH diameter, the greater the retinal displacement, which contributes to greater residual sensation of metamorphopsia after surgery. This was a retrospective study, and the number of clinical cases was small. Another limitation of this study was that the extent of ILM peeling was not measured. In the future, it will be necessary to increase the number of cases and further elucidate the surgical techniques related to metamorphopsia in the pathology of MH.
