# Correction to: Retinal displacement using optical coherence tomography angiography and metamorphopsia in eyes undergoing macular hole surgery

**DOI:** 10.1007/s10384-025-01247-7

**Published:** 2025-07-10

**Authors:** Shun Tsukahara, Asuka Takeyama, Masahiro Ishida

**Affiliations:** 1 Department of Ophthalmology, Toho University Graduate School of Medicine, Tokyo, Japan; 2 Department of Ophthalmology, Toho University Ohashi Medical Center, Ohashi, 2‑22‑36, Meguro‑ku, Tokyo, 153‑8515 Japan

**Correction to: Japanese Journal of Ophthalmology (2025) 69:395–402** 10.1007/s10384-025-01176-5

In Table 1 of this article, third to eighth entries 3MNV%, 3MFV%, 3MNV%, 3MFV%, 3MNV% and 3MFV% in the fourth column were incorrect and should have been 3MNH%, 3MFH%, 6MNV%, 6MFV%, 6MNH% and 6MFH%.

**Table 1:** Comparison of retinal displacement% in near and far regions

	Retinal displacement%	*P* value		Retinal displacement%	*P* value
2wNV%	–28.80±8.59	*P*<0.001	3MNV%	–23.25±8.29	*P*<0.001
2wFV%	–12.72±13.82		3MFV%	–5.31±3.93	
2wNH%	–19.43±15.48	*P*<0.001	3MNH%	–17.06±11.72	*P*<0.001
2wFH%	–5.97±4.13		3MFH%	–6.32±7.77	
1MNV%	–24.08±7.77	*P*<0.001	6MNV%	–22.45±8.47	*P*<0.001
1MFV%	–5.85±3.47		6MFV%	–7.51±9.20	
1MNH%	–17.59±17.44	*P*<0.001	6MNH%	–15.82±12.54	*P*<0.001
1MFH%	–4.85±4.99		6MFH%	–4.68±4.82	

Paired t-tests were used to determine the significance

*NV%*: near-vertical retinal displacement%, *NH%*: near-horizontal retinal displacement%

*FV%*: far-vertical retinal displacement%, *FH%*: far-horizontal retinal displacement%

The updated Table 1 is given in this Correction.

